# Interpreting high levels of unfolded Von Willebrand Factor in patients with the antiphospholipid syndrome

**DOI:** 10.3389/fimmu.2024.1514433

**Published:** 2024-12-12

**Authors:** Romy de Laat-Kremers, Shengshi Huang, Hugo ten Cate, Marisa Ninivaggi, Bas de Laat, Katrien Devreese

**Affiliations:** ^1^ Department of Data Analysis and Artificial Intelligence, Synapse Research Institute, Maastricht, Netherlands; ^2^ Department of Functional Coagulation, Synapse Research Institute, Maastricht, Netherlands; ^3^ Department of Internal Medicine and Biochemistry, Maastricht University Medical Center, Maastricht, Netherlands; ^4^ Cardiovascular Research Institute Maastricht (CARIM), Maastricht University, Maastricht, Netherlands; ^5^ Coagulation Laboratory, Department of Laboratory Medicine, Ghent University Hospital, Ghent, Belgium; ^6^ Department of Diagnostic Sciences, Ghent University, Ghent, Belgium

**Keywords:** Von Willbrand Factor, antiphospholipid syndrome, thrombosis, unfolded VWF, VWF pro-peptide

## Abstract

**Introduction:**

Unfolded Von Willebrand Factor (VWF) is increased in thrombotic pathologies such as myocardial infarction. Unfolded VWF mediates the binding of platelets without the need for collagen. β_2_-glycoprotein I (β_2_-GPI) is a natural inhibitor of the platelet-VWF interaction. The antiphospholipid syndrome (APS) is associated with thrombosis, with an important pathophysiological role of auto-antibodies directed against β_2_-GPI.

**Methods:**

(Unfolded) VWF levels were studied in normal controls (n=93), APS patients (n=64), non-APS thrombosis patients (n=39) and non-APS auto-immune disease (AID) patients (n=49.

**Results:**

Unfolded VWF levels were respectively, 53%, 50% and 36% higher in APS patients, non-APS thrombosis patients and AID patients, compared to normal controls (p<0.0001). Unfolded VWF levels above the 90^th^ percentile in normal controls were associated with an odds of APS (OR: 8.51; CI:3.26 - 22.2; p<0.001), compared to ORs of non-APS thrombosis (OR:5.87; CI:2.07 - 16.7, p=0.001) and AID (OR:3.71; CI:1.40 – 9.87; p=0.009).

**Discussion:**

We found that APS patients have high levels of unfolded VWF in their circulation. In APS, auto-antibodies against-β2-GPI may interfere with the β2-GPI-mediated inhibition of VWF-platelet interaction. Therefore, the higher unfolded VWF levels in APS could in part explain the association of APS and thrombotic complications.

## Highlights

- Unfolding of VWF exposes the binding site for platelet VWF receptor glycoprotein Ib.
- β2-GPI binds to unfolded VWF (uVWF) and inhibits platelet binding.
- The antiphospholipid syndrome (APS) is associated with anti-β_2_-GPI auto-antibodies.
- Antibodies against β2-GPI counteracting the inhibition are associated with thrombosis.
- uVWF levels > 90^th^ percentile were associated with a 8.5-fold higher OR for APS.

## Introduction

The hemostatic system is a highly regulated process to avoid blood loss upon injury, and at the same time preventing unwanted intravascular clot formation which causes thrombotic complications. Von Willebrand factor (VWF) is a multimeric plasma protein that regulates the adhesion of platelets to subendothelial collagen at sites of vascular injury (1). Unfolding of VWF exposes the binding site for platelet VWF receptor glycoprotein Ib. The unfolding of VWF is one step in the cross-talk between the coagulation system and platelets, and is tightly regulated to prevent unwanted platelet adherence to the vascular wall under physiological conditions (2). In general, higher VWF levels are associated with a more pro-coagulant state, especially as VWF serves as a carrier for procoagulant factor VIII in the blood stream. As a results, higher VWF levels are associated with (unprovoked) venous thromboembolism and arterial thrombosis (3–6).

In Von Willebrand Disease (VWD) Type 2B, patients suffer from a gain of function defect in VWF, leading to spontaneous platelet binding and rapid clearance of platelets and large VWF multimers (7–9) Subsequently, VWD Type 2 patients lack high molecular weight VWF multimers and suffer from thrombocytopenia, which results in a bleeding phenotype (10) On the contrary, in thrombotic thrombocytopenia purpura (TTP), the activity of VWF cleaving enzyme ADAMTS-13 is low or absent, leading to an excess of ultra-large VWF multimers in the circulation (7–9, 11) These ultra-large VWF multimers cause spontaneous platelet binding and subsequent thrombosis in the microvasculature.

Another condition associated with thrombocytopenia and the formation of microthrombi in the vasculature is the antiphospholipid syndrome (APS) (8) APS is an auto-immune disease associated with thrombosis and/or recurrent pregnancy loss in combination with the persistent presence of auto-antibodies directed to β2-glycoprotein I (β2GPI), amongst others (7). Interestingly, VWF is an acute phase reactant and is subsequently elevated during an inflammatory response and in auto-immune diseases (8). Furthermore, VWF has been implicated in other pathologies, such as endothelial dysfunction, cancer (metastasis) (7, 8, 12, 13).

Especially anti-β2-GPI antibodies that express lupus anticoagulant (LAC) activity are associated with thrombosis in APS (13). APS patients with β2-GPI-dependent LAC are reported to have higher levels of the unfolded form of VWF (7, 12, 13) We have previously shown that anti-β2-GPI antibodies do not increase the expression of active VWF, but inhibit the antithrombotic capacity of β2-GPI. When VWF unfolds, the A1 domain, which is responsible for platelet binding via GPIb, is exposed. β2-GPI in its open conformation shields off the A1 domain thereby inhibits and modulates platelet binding to VWF and subsequently thrombus formation. As a result, unfolded VWF will always be generated but β2-GPI, among others, regulates the process of platelet binding. Subsequently, auto-antibodies against β2-GPI that counteract this inhibitory action contribute to the increased risk of arterial thrombosis in APS (8).

The determination of total VWF levels is routinely performed in clinical laboratories. However, *in vivo* VWF first needs to unfold (hence unfolded VWF) to bind to platelets, in which the VWF A1 domain is in the GPIb binding conformation (11) In a research setting, an immunosorbent assay was developed specifically for the measurement of unfolded VWF (11) This assay has shown its clinical applicability in VWD Type 2B, TTP, hemolysis, elevated liver enzymes and low platelets (HELLP) syndrome, systemic inflammatory response syndrome (SIRS), sickle cell disease, and myocardial infarction (11, 13–16).

In this study, we investigated whether high plasma levels of unfolded VWF are associated with thrombotic APS, non-APS thrombosis and non-APS auto-immune diseases (AID).

## Materials and methods

### Study population

APS, thrombosis and auto-immune disease (AID) patient and normal controls were a subset of the APS multicenter study, for which patients and controls were enrolled at multiple medical European centers (17) The research was approved by the local institutional review boards and ethics committee of Ghent University Hospital, and the study was performed in accordance with the Declaration of Helsinki.

Thrombotic APS patients were included if APS was diagnosed according to the updated Sapporo criteria clinical and ISTH-SSC laboratory criteria (18, 19), and thrombosis was documented as a clinical criterion. Normal controls were defined as patients tested for antiphospholipid antibodies (aPL) (lupus anticoagulant (LAC), anticardiolipin (aCL) and anti-β2GPI IgG/IgM antibodies), visiting the hospital for (non)criteria clinical symptoms of APS, such as subfertility or an incidentally prolonged activated partial thromboplastin time (aPTT). Thrombosis patients tested negative for aPL antibodies; auto-immune disease patients did not fulfill the clinical criteria for APS (17). AID patients and normal controls did not have a history of thrombosis, and APS and thrombosis patients did not have active thrombotic complications at the time of sample collection. Subjects below the age of 18 years and subjects using oral contraceptives were excluded. The study population consisted of 54 thrombotic APS patients, 39 non-APS thrombosis patients and 49 non-APS auto-immune disease (AID) patients and 93 normal controls. Blood was collected on 3.2% citrate in a 9:1 ratio and platelet poor plasma (PPP) was prepared by centrifuging twice at 2821g for 10 minutes. PPP was stored at -80°C until further use.

### Determination of total and unfolded VWF, and VWF pro-peptide

Total VWF antigen levels were determined using the Liatest VWF assay on an automated STA-R Max coagulation analyzer (Diagnostica Stago, France). Unfolded VWF levels were measured using a llama-derived single-domain antibody (VHH) directed against a cryptic epitope in the A1 domain of VWF, which is only exposed upon unfolding of VWF, as previously described (20). VWF pro-peptide (VWFpp) was measured using the anti-human VWFpp MW1939 antibody pair and Tool Set 2 (Sanquin, The Netherlands) according to the manufacturer instructions.

### Testing for aPL

The presence of aPL was defined as positivity for LAC and/or aCL and/or antiβ2-GPI IgG/IgM (18, 21) LAC was assessed according to the three-steps guidelines of the Subcommitee on LAC of the International Society on Thrombosis and Haemostasis (ISTH) using an aPTT and a dilute Russel’s Viper VenomTime (dRVVT) screen and confirm reagents (21) aCL, β2-GPI, and anti-phosphatidylserine/prothrombin (anti-PS/PT) antibodies (IgG and IgM were detected using the QUANTA Lite ELISA (Inova Diagnostics, USA) according to the manufacturer’s instructions (22).

### Statistics

Statistical analysis was performed in GraphPad Prism (version 10.0.1, GraphPad Software, USA) and the Statistical Package for the Social Sciences (SPSS; version 27, IBM, USA). The Mann-Whitney test and Kruskal-Wallis test were used to test for differences between groups, depending on the number of groups in the analysis. For categorical variables, the χ^2^ test was used. 90^th^ percentile cut off points were determined in normal controls, and APS, thrombosis and AID patient groups were stratified accordingly. Multinomial logistic regression was used to study the association between high VWF levels and the odds of suffering for APS, thrombosis or AID. Models were further adjusted for age and sex. P-values below 0.05 were considered statistically significant.

## Results

We measured the levels of total VWF, unfolded VWF and VWF pro-peptide in a cohort of 93 normal controls, 54 thrombotic APS patients, 39 non-APS thrombosis patients and 49 non-APS AID patients (**Table 1**).

**Table 1:** General characteristics of thrombotic APS patients and control subjects.

	Normal controls	APS patients	Thrombosis patients	AID patients
N	93	54	39	49
Age (years)	37 ± 7	48 ± 14	47 ± 13	46 ± 13
Sex, n (% female)	88 (95%)	36 (64%)	22 (56%)	42 (86%)
Primary APS, n (%)	N/A	52 (96%)	N/A	N/A
Secondary APS, n (%)	N/A	2 (4%)	N/A	N/A
Lupus Anticoagulant, n (%)	N/A	38 (70%)	0 (0%)	2 (4%)
anti-cardiolipin antibodies, n (%)	N/A	32 (59%)	0 (0%)	4 (8%)
anti-β2GPI antibodies, n (%)	N/A	34 (63%)	0 (0%)	5 (10%)
anti-PS/PT, n (%)	N/A	32 (59%)	3 (8%)	6 (12%)
Thrombosis, n (%)	0 (0%)	54 (100%)	39 (100%)	0 (0%)
Arterial thrombosis, n (%)	0 (0%)	22 (41%)	7 (18%)	0 (0%)
Venous thrombosis, n (%)	0 (0%)	40 (74%)	32 (82%)	0 (0%)

Age is shown as mean ± standard deviation and all other variables are shown as the number of subjects with the percentage of subjects between brackets. AID, auto-immune disease; APS, antiphospholipid syndrome.

Controls and APS patients were predominantly female, whereas thrombosis patients and auto-immune disease patients were more often male (p<0.001). Moreover, patients were significantly older compared to normal controls (p<0.001). Specifically for APS patients, 96% suffered from primary APS and 4% from secondary APS. LAC was present in 70% of the APS patients, whereas 59% and 63%, respectively, were positive for aCL or anti-β2GPI antibodies. Additionally, anti-PS/PT antibodies were detected in 59% of APS patients. As thrombosis was an inclusion criterium for APS patients and thrombosis patients, 100% of APS patients and thrombosis patients experienced thrombosis. In 74% of APS patients, thrombosis presented as venous thrombosis and in 41% as arterial thrombosis. In thrombosis patients, 82% experienced venous thrombosis and 18% arterial thrombosis. Thrombosis was an exclusion criterium for normal control and AID patient groups, and therefore, none of the normal and AID control subjects experienced prior thrombosis.

**Figure 1:**
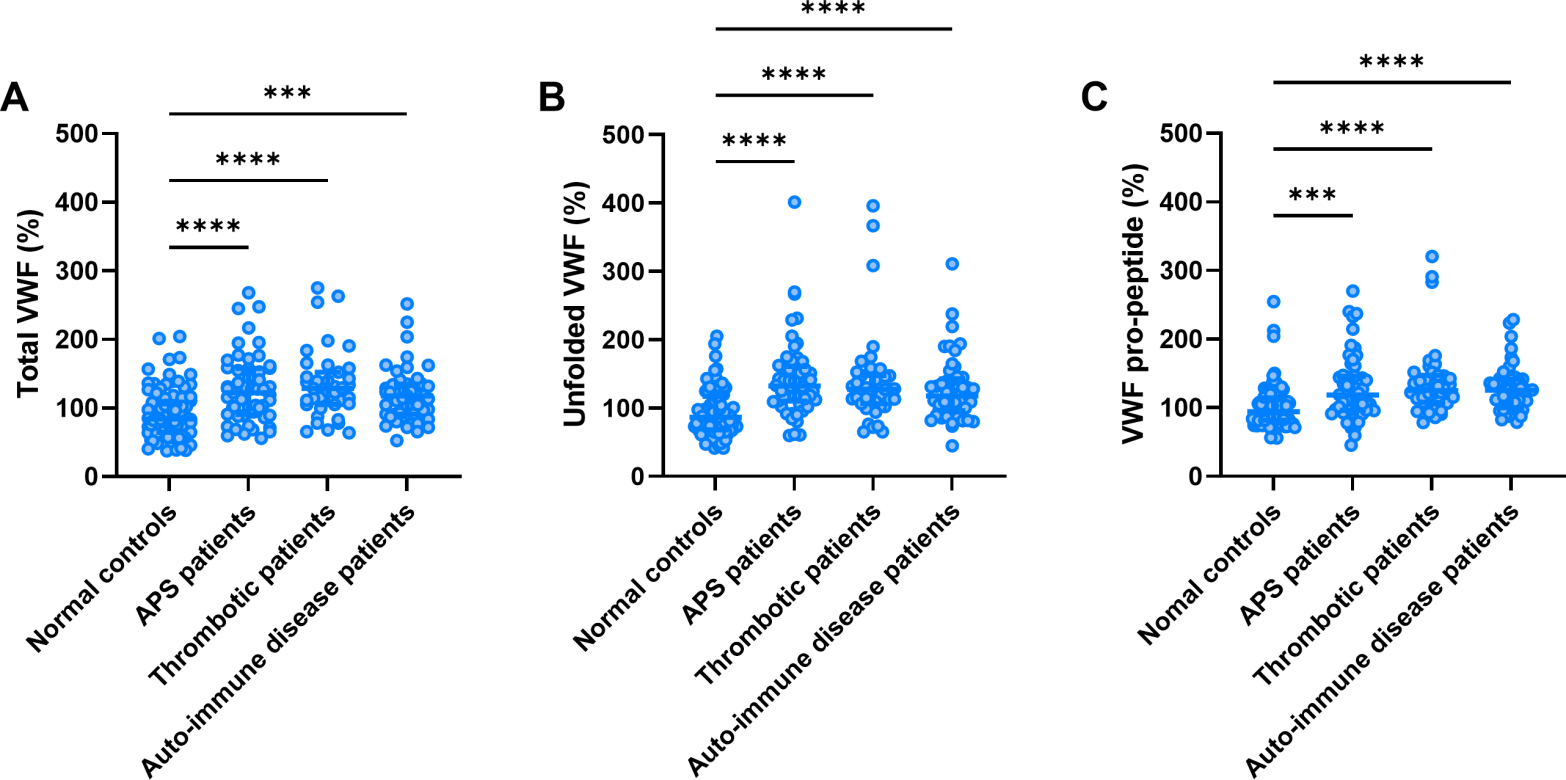
Total VWF, unfolded VWF and VWF pro-peptide levels. Total VWF **(A)**, unfolded VWF **(B)** and VWF pro-peptide levels **(C)** in thrombotic APS, thrombosis and auto-immune disease (AID) patients and normal controls. ***, **** indicate a statistically significant difference with a p-value below 0.001 and 0.0001, respectively.

Total VWF levels were significantly higher in APS patients, thrombosis patients and AID patients compared to normal controls (+41%, +49% and +31%, respectively; **Figure 1A**). Unfolded VWF levels were significantly higher in APS (+53%), thrombosis (+50%), and AID patients (+36%; **Figure 1B**). Similarly, VWF pro-peptide levels were higher in APS (+27%), thrombosis (+36%), and AID patients (+26%; **Figure 1C**).

**Figure 2:**
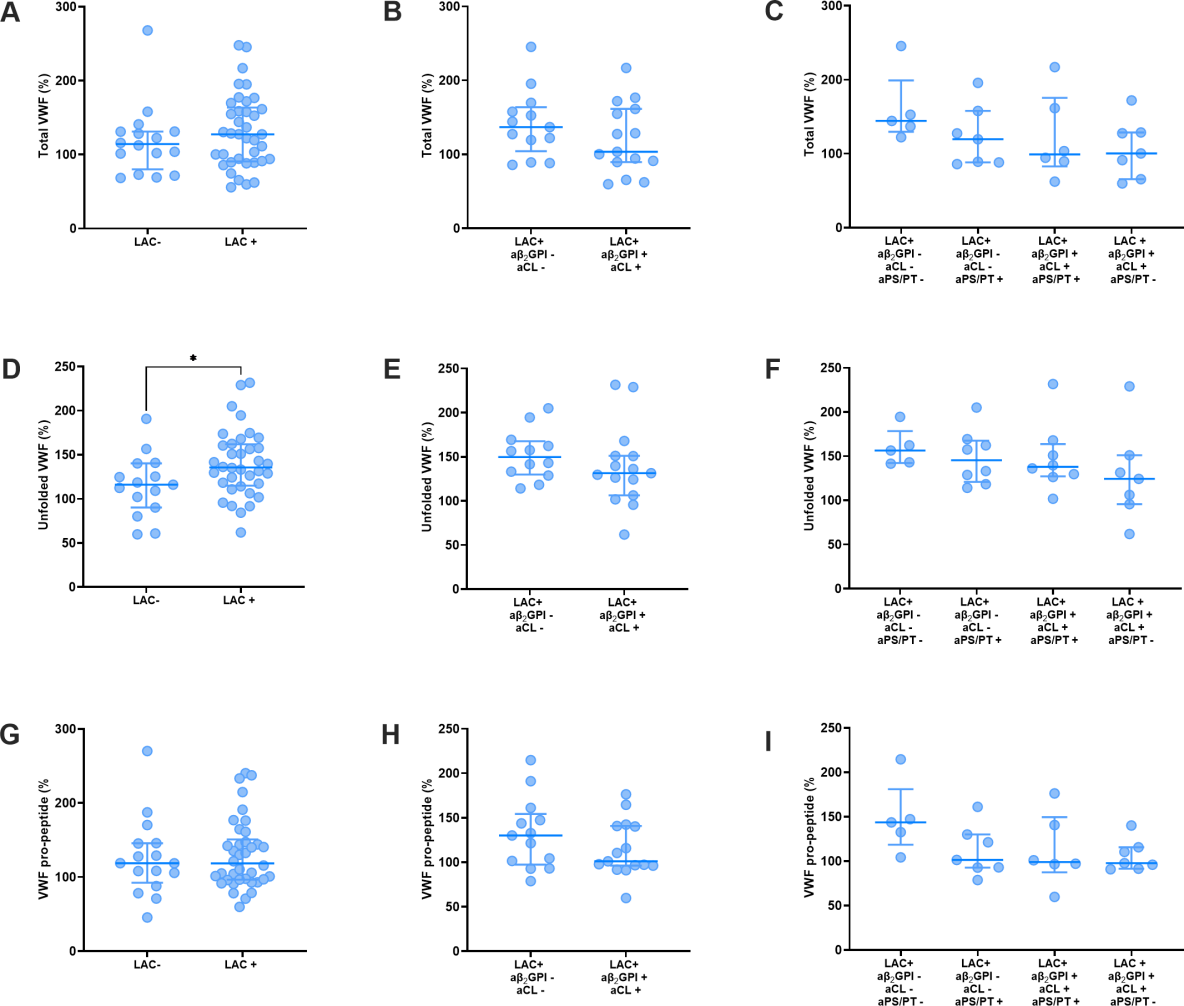
VWF levels in thrombotic APS patients stratified for lupus anticoagulant status and antibody profile. **(A–C)** Total VWF levels stratified according to lupus anticoagulant (LAC) status **(A)**, anti-β_2_GPI and anti-cardiolipin status **(B)** and anti-PS/PT status of APS patients **(C)**. **(D–F)** Unfolded VWF levels stratified according to lupus anticoagulant (LAC) status **(D)**, anti-β_2_GPI and anti-cardiolipin status **(E)** and anti-PS/PT status of APS patients **(F)**. **(G–I)** VWF pro-peptide levels stratified according to lupus anticoagulant (LAC) status **(G)**, anti-β_2_GPI and anti-cardiolipin status **(H)** and anti-PS/PT status of APS patients **(I)**. Data is represented as individual values with lines indicating the median and interquartile range. * Indicates a statistically significant difference with a p-value below 0.05.

Stratification of APS patients by LAC status revealed that unfolded VWF levels were higher in patients with LAC (**Figure 2D**), whereas VWF and VWF pro-peptide did not differ between patients with and without LAC (**Figures 2A, G**). Total VWF, unfolded VWF and VWF pro-peptide did not differ significantly between LAC positive APS patients with or without antibodies against β_2_GPI and cardiolipin (**Figures 2B, E, H**). Furthermore, the presence of anti-PS-PT antibodies in LAC positive APS patients with or without antibodies against β_2_GPI and cardiolipin did not significantly affect the levels of total VWF (**Figure 2C**), unfolded VWF (**Figure 2F**), and VWF pro-peptide (**Figure 2I**).

A cut-off value was calculated as the 90^th^ percentile of the normal controls for total VWF (138%), unfolded VWF (136%) and VWF pro-peptide (136%). Patients were classified based on the determined cut-off values. For total VWF, 33% of APS patients, 36% of thrombosis patients, and 20% of AID patients were above the cut-off value (**Table 2**). For unfolded VWF, 46% of APS patients, 36% of thrombosis patients, and 29% of AID patients were above the cut-off value. Lastly, 39% of APS patients, 41% of thrombosis patients, and 41% of AID patients were above the cut-off value for VWF pro-peptide.

**Table 2:** Odds ratio for APS, thrombosis or AID at VWF levels above the 90^th^ percentile.

			Model 1		Model 2	
	≤ 90th percentile	> 90th percentile	OR (95% CI)	*p-value*	OR (95% CI)	*p-value*
Total VWF						
Normal controls, n (%)	84 (90.3%)	9 (9.7%)	-1-		-1-	
Antiphospholipid syndrome, n (%)	36 (66.7%)	18 (33.3%)	4.67 (1.92 – 11.3)	*<0.001*	3.82 (1.44 – 10.1)	*0.007*
Thrombosis, n (%)	25 (64.1%)	14 (35.9%)	5.23 (2.02 - 13.5)	*<0.001*	4.65 (1.64 – 13.2)	*0.004*
Auto-immune disease, n (%)	39 (79.6%)	10 (20.4%)	2.39 (0.91 - 6.36)	*n.s.*	1.92 (0.68 – 5.37)	*n.s*
Unfolded VWF						
Normal controls, n (%)	84 (90.3%)	9 (9.7%)	-1-		-1-	
Antiphospholipid syndrome, n (%)	29 (53.7%)	25 (46.3%)	8.05 (3.37 - 19.2)	*<0.001*	8.51 (3.26 - 22.2)	*0.001*
Thrombosis, n (%)	25 (64.1%)	14 (35.9%)	5.23 (2.02 - 13.5)	*0.001*	5.87 (2.07 - 16.7)	*0.001*
Auto-immune disease, n (%)	35(71.4%)	14 (28.6%)	3.73 (1.48 - 9.42)	*0.005*	3.71 (1.40 – 9.87)	*0.009*
VWF pro-peptide						
Normal controls, n (%)	84 (90.3%)	9 (9.7%)	-1-		-1-	
Antiphospholipid syndrome, n (%)	33 (61.1%)	21 (38.9%)	5.94 (2.47 – 14.3)	*<0.001*	5.19 (1.96 – 13.7)	*0.001*
Thrombosis, n (%)	23 (59.0%)	16 (41.0%)	6.49 (2.54 - 16.6)	*<0.001*	6.21 (2.20 – 17.5)	*<0.001*
Auto-immune disease, n (%)	29 (59.2%)	20 (40.8%)	6.44 (2.64 - 15.7)	*<0.001*	5.70 (2.21 – 14.7)	*<0.001*

Model 1: Crude; Model 2: adjusted for age and sex. OR, Odds ratio; CI, confidence interval; VWF, Von Willebrand Factor; VWF Ag; VWF Antigen; VWFpp, VWF pro-peptide.

We performed multinomial logistic regression to investigate whether the high levels of total VWF, unfolded VWF and/or VWF pro-peptide above the cut-off value are associated with APS, non-APS thrombosis and/or non-APS AID (**Table 2**). In the crude model, total VWF values above the 90^th^ percentile cut-off resulted in an odds ratio (OR) of 4.67 (CI: 1.92-11.3, p<0.001) for APS, and an OR of 5.23 (CI: 2.02-13.5, p<0.001) for non-APS thrombosis. After adjustment for age and sex, high total VWF gave an OR of 3.82 (CI: CI: 1.44-11.3, p<0.001) for APS, an OR of 4.65 (CI: 1.64-13.2, p<0.001) for non-APS thrombosis. No significant association was detected between high total VWF levels and AID.

**Figure 3:**
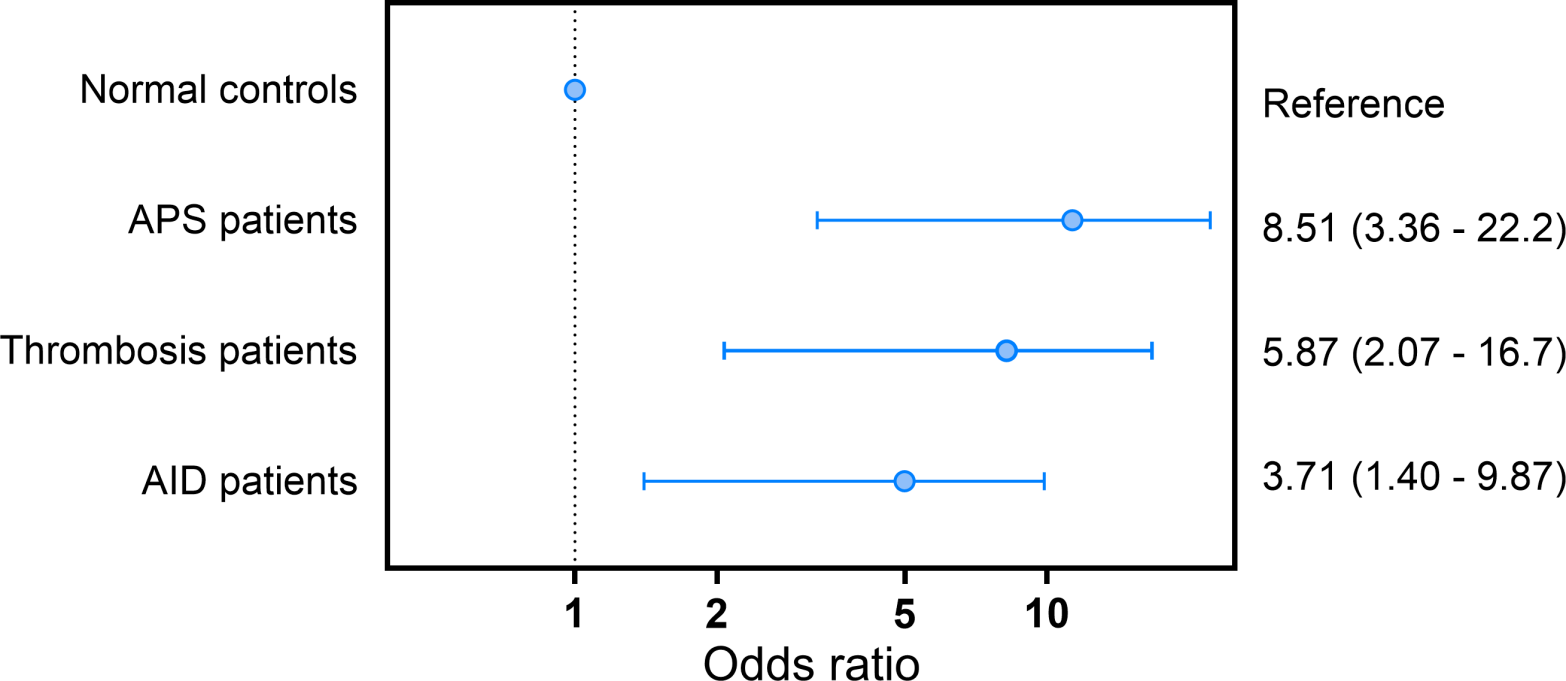
Odds ratio for APS, thrombosis and AID in subjects with unfolded VWF levels above the 90^th^ percentile cut-off value.

Unfolded VWF levels above the 90^th^ percentile were significantly associated with APS (OR: 8.0; CI: 3.37 – 19.2; p<0.001), non-APS thrombosis (OR: 5.23; CI:2.02 - 13.5; p=0.001) and AID (OR:3.73; CI: 1.48 - 9.42; p=0.005) in the crude model (**Table 2**). Adjusting for age and sex strengthened the association between high unfolded VWF levels and APS (OR: 8.51; CI:3.26 - 22.2; p<0.001), thrombosis (OR:5.87; CI:2.07 - 16.7, p=0.001) and AID (OR:3.71; CI:1.40 – 9.87; p=0.009; **Figure 3**).

VWF pro-peptide levels above the 90^th^ percentile were significantly associated with APS, thrombosis and AID in the crude model (OR: 5.94, OR: 6.49, and OR 6.44, respectively; all p<0.001) and in the fully adjusted model (OR:5.19, OR:6.21, and OR:5.70; p<0.001; **Table 2**). Furthermore, we investigated whether subjects with two or three VWF levels above the 90^th^ percentile of control subjects have higher odds of having APS, thrombosis or AID (**Supplementary Table 1**). The odd ratio for APS for high unfolded VWF levels alone was higher than a combination of two or more VWF levels above the 90^th^ percentile.

## Discussion

In this study, we found a significant association between APS and unfolded VWF levels, total VWF levels and VWF pro-peptide levels. APS is an auto-immune disorder that is typically associated with thrombotic events and pregnancy morbidity, in addition to the presence of aPL that bind to cofactors, of which β_2_GPI and prothrombin are the most important ones (7). Total VWF and VWF pro-peptide levels were significantly higher in APS, thrombosis controls and AID patients compared to normal controls, indicating that more VWF is released from the vessel wall and VWF is unfolded more frequently (1). Edvardsen et al. reported that elevated levels of VWF are significantly associated with venous thromboembolism (VTE), especially unprovoked deep venous thrombosis (4), which is in line with our current findings. Moreover, VWF has been proposed as an independent risk factor for VTE (3, 5), and several studies have found associations between high VWF levels and arterial thrombosis, especially in subjects with coronary heart disease (6). In addition to its role in coagulation, VWF is known to be implicated in physiological and pathological processes, including angiogenesis smooth muscle cell proliferation and the maintenance of endothelial function (23). Furthermore, animal studies have shown that decreased VWF levels are associated with slower progression of atherosclerosis, which is in turn associated with a reduced risk of thrombosis (23, 24).

In the studied cohort, unfolded VWF was increased in APS, thrombosis and AID patients, confirming previous findings of Hulstein and al (12). The transformation of VWF into its unfolded form is a crucial step for VWF-platelet interaction, because it exposes the binding site for the platelet VWF receptor GPIb in the VWF A1 domain (25). If VWF unfolding is disturbed under pathophysiological circumstances, unfolded VWF circulates in the blood in the absence of vascular injury (26). High circulating levels of unfolded VWF are known to be associated with thrombosis, e.g. in TTP, sickle cell disease, and myocardial infarction (3–6). Unfolded VWF can be inhibited in the bloodstream by β_2_-GPI, which binds to the A1 domain of unfolded VWF and thereby hinders the binding of platelets (7, 27). However, in APS, auto-antibodies against-β_2_-GPI interfere with the β_2_-GPI-mediated inhibition of the VWF-platelet interaction (12). Subsequently, auto-antibodies against β_2_-GPI are associated with the development of thrombotic events (7, 8). In the current study, we found a remarkable difference in ORs for the association of high unfolded VWF levels and APS compared to non-APS thrombosis patients and non-APS AID patients. Subjects with unfolded VWF levels above the 90^th^ percentile cut-off value had a 8.5-fold higher OR for APS. Interestingly, high unfolded VWF levels were significantly associated with non-APS thrombosis as well, although with a lower OR of 5.9. For non-APS, non-thrombosis AID a 3.7-fold higher OR was found. These data may point to an additive effect of the auto-immune disease component and the thrombosis component of the APS pathology, resulting in a stronger association of high unfolded VWF levels and APS, although this needs to be confirmed in other populations.

A potential stimulatory effect of aPL on VWF secretion has been described, resulting in higher VWF levels in patients with systemic lupus erythematosus and APS (27). Our results suggest that indeed VWF is a biomarker for not only APS, but also auto-immune diseases and thrombosis in general. Especially the unfolded form of VWF is strongly associated with APS, indicating that the pathophysiological process underlying this finding is indeed in part determined by effects in the unfolding and inactivation process of VWF.

This study has several limitations. One limitation is the smaller size of the patient cohort, although the number of patients was sufficient for statistically meaningful analyses. APS is a relatively uncommon condition, and therefore it is difficult to collect plasma from a large number of patients. In addition, the cohort shows a significant difference in age- and sex-distribution between the patient and control groups as APS and AID are more common in women. Another limitation is the wide confidence intervals for the ORs of the associations between APS and VWF levels, albeit that the association was statistically significant. Moreover, the number of subjects in each antibody specific profile group was not sufficient to detect statistically different unfolded VWF levels between these groups. In addition, due to the study setup, we were unable to differentiate between the effect of medication administered to patients and the crude effect the diseases itself.

In conclusion, we found a significant association between unfolded VWF levels above the 90^th^ percentile cut-off value and the occurrence of APS and thrombosis in general. High unfolded VWF levels resulted in a 8.5-fold higher odds ratio for APS in this cohort. Further studies are required to investigate the precise biological mechanism of action of elevated unfolded VWF in patients with auto-immune diseases. Larger prospective cohort studies are required to determine whether (unfolded) VWF can serve as a biomarker for thrombotic APS for patients suspected of suffering from APS.

## Data Availability

The original contributions presented in the study are included in the article/**Supplementary Material**. Further inquiries can be directed to the corresponding author.
