# Supplementary material for: Interpreting high levels of unfolded Von Willebrand Factor in patients with the antiphospholipid syndrome
Source: Front Immunol. 2024 Dec 12;15:1514433. doi: 10.3389/fimmu.2024.1514433 (PMC11669600; doi:10.3389/fimmu.2024.1514433)
Supplement: Supplementary file 1 [file Table1.docx]

Interpreting High Levels of Unfolded Von Willebrand Factor in Patients with the Antiphospholipid Syndrome

**Authors**: Romy de Laat-Kremers^1*^, Shengshi Huang^2,4*^, Hugo ten Cate^3^,^4^, Marisa Ninivaggi^2^, Bas de Laat^1,2^†, Katrien Devreese^5,6^†

^1^ Department of Data Analysis and Artificial Intelligence, Synapse Research Institute, Maastricht, the Netherlands;

^2^ Department of Functional Coagulation, Synapse Research Institute, Maastricht, the Netherlands

^3^ CARIM, Maastricht University, Maastricht, the Netherlands;

^4^ Departments of Internal medicine and Biochemistry, Maastricht University Medical Center, Maastricht, the Netherlands

^5^ Coagulation Laboratory, Department of Laboratory Medicine, Ghent University Hospital, Ghent, Belgium;

^6^ Department of Diagnostic Sciences, Ghent University, Ghent, Belgium;

*,† Equal contribution

**Corresponding author:**

Name: Romy de Laat - Kremers

Address: Pastoor Habetsstraat 50, 6217KM, Maastricht

E-mail address: r.delaat@thrombin.org

Phone: 0031 618417835

# Supplementary data

**Supplementary Table 1: Odds ratio of APS diagnosis due to combinations of high VWF levels**

|  |  |  |  | **Model 1** | |  | **Model 2** | |
| --- | --- | --- | --- | --- | --- | --- | --- | --- |
|  |  | **≤ 90th percentile** | **>90th percentile** | **OR (95% CI)** | ***p-value*** |  | **OR (95% CI)** | ***p-value*** |
| **Two levels >90th percentile** | |  |  |  |  |  |  |  |
|  | Normal controls, n (%) | 86 (92.5 %) | 7 (7.5 %) | -1- |  |  | -1- |  |
|  | Antiphospholipid syndrome, n (%) | 33 (61.1 %) | 21 (38.9 %) | 7.82 (3.04 – 20.1) | *<0.001* |  | 6.86 (2.46 – 19.1) | *<0.001* |
|  | Thrombosis, n (%) | 25 (64.1 %) | 14 (35.9 %) | 6.88 (2.50 – 18.9) | *<0.001* |  | 6.49 (2.16 – 19.5) | *0.001* |
|  | Auto-immune disease, n (%) | 36 (73.5 %) | 13 (26.5 %) | 4.44 (1.64 – 12.0) | *0.003* |  | 3.77 (1.32 – 10.8) | *0.003* |
| **Three levels >90th percentile** | |  |  |  |  |  |  |  |
|  | Normal controls, n (%) | 89 (95.7 %) | 4 (4.3 %) | -1- |  |  | -1- |  |
|  | Antiphospholipid syndrome, n (%) | 41 (75.9 %) | 13 (24.1 %) | 7.06 (2.17 - 22.9) | *0.001* |  | 5.88 (1.63 – 21.1) | *0.007* |
|  | Thrombosis, n (%) | 31 (79.5 %) | 8 (20.5 %) | 5.74 (1.62 – 20.4) | *0.007* |  | 5.21 (1.32 – 20.6) | *0.019* |
|  | Auto-immune disease, n (%) | 43 (87.8 %) | 6 (12.2 %) | 3.11 (0.83 – 11.6) | *n.s.* |  | 2.50 (0.63 – 9.95) | *n.s.* |

**Model 1:** Crude; **Model 2**: adjusted for age and sex. Abbreviations: OR: Odds ratio; CI: confidence interval; VWF: Von Willebrand Factor.
